# 肺部微小病灶术前定位方法的进展及新思路

**DOI:** 10.3779/j.issn.1009-3419.2012.06.10

**Published:** 2012-06-20

**Affiliations:** 200030 上海，上海交通大学附属胸科医院，上海市肺部肿瘤临床医学中心胸外科 Department of Thoracic Cancer, Shanghai Chest Hospital, Shanghai Lung Tumor Clinical Medical Center, Shanghai 200030, China

**Keywords:** 肺部微小病灶, 术前定位, CT模拟机, Pulmonary tiny lesions, Preoperative localization, CT analog machine

## Abstract

肺部周围型微小病灶在手术中的定位比较困难。现有各种术前定位方法依然存在缺陷，应用有限，难以适应临床微小病灶日益增多的迫切需要。本文拟就目前的相关进展予以综述，并简介我们近期探索尝试的新方法。

## Abstract (en)

Pulmonary peripheral tiny lesions are difficult to be localized during operation. The current each preoperative localization methods still exist defects and limited to application which can not adapt to urgent need for increasing numbers of the clinical pulmonary tiny lesions. This paper intends to summarize the present relevant progress, and introduce our recent attempt to explore a new method.

近年来，低剂量螺旋计算机断层扫描（computed comography, CT）重建技术有了长足进步并日趋成熟。随着国家经济发展和人民健康意识的提升，常规体检率逐年增加，肺部微小病灶的检出量日趋增多。肺部微小病灶包括直径 < 20 mm的孤立性肺结节（solitary pulmonary nodule, SPN）、肺磨玻璃样病变（ground glass opacity, GGO）等。胸外科医师对此类病变十分重视，原因是其中包含相当比例的早期肺癌。据2007年ACCP临床实践指南报道^[1]^：肺部孤立性结节的检出率为8%-51%，其中1.1%-12%为恶性；而纯型磨玻璃样病变（pure ground glass opacity, pGGO）则有高达59%-73%的恶性率。理论上，对于周围型肺部微小病灶，应当首先局部手术切除，快速冰冻病理定性，再酌情决定适宜的进一步手术范围。但在实际手术操作中，如何定位肉眼难以察及或手指不能触知的肺内周围型微小病灶，是攸关手术方式的关键所在。极端情况下只能行肺叶切除以免遗漏病灶，但这势必会牺牲良性病变患者正常的肺组织，如何减少或避免此种尴尬现象，已成为亟待解决的难题。为此，本文拟对肺部微小病灶的定位方法及目前的研究现状^[2]^进行综述，并简介我们近期探索尝试的新方法。

## 1 定位方法的历史回顾及研究现状

### 1.1 术前定位法

术前定位法包括CT引导金属钩定位、注射亚甲蓝染色、注射胶原或琼脂溶胶定位及注射放射性核素示踪等方法。

#### 1.1.1 金属钩穿刺法

1992年Plunkett等^[3]^根据金属钩对乳腺小病灶定位技术的原理，开始进行在CT下用金属钩对肺部周围型结节定位的研究和实践。金属钩（Hookwire）是一根细长的前端弯曲成钩状的金属丝，在CT扫描（层厚3 mm）定位后套针穿刺进入，CT引导下经皮穿刺到病灶或其附近后，将金属丝释放并回收套针，其前端展开成钩状，起到固定作用。重复CT扫描显示钩子膨胀打开并位于病灶或者附近肺组织内（距离病灶边缘 < 5 mm）。剪断金属线，并立即送往手术室在胸腔镜下沿金属丝周围行肺楔形切除术^[4-7]^。此定位技术准确率较高，但当病灶距脏层胸膜较近时肺萎陷后金属钩较容易发生移位导致定位失败，其它的并发症主要包括气胸、胸痛、咯血及胸膜反应等^[8]^，个别病例曾出现断针残留。另外，此方法对患者及操作人员的辐射较多，在一定程度上也会增加患者的心理负担。同时，过程中要求CT室和手术室密切配合，确保定位后立即进行手术，这种运作的方式在繁忙的医院也有一定的困难。

#### 1.1.2 亚甲蓝染色法

1994年，Lenglinger等^[9]^及Wicky等^[10, 11]^术前在CT引导下，使用穿刺针经皮穿刺到肺内病灶，然后注射适量的亚甲蓝，对病灶、针道和脏层胸膜表面进行染色，随即进行胸腔镜或开胸手术，术中根据胸腔镜或肉眼观察到的肺表面染色范围，行楔形切除，均获得了比较满意的效果。此方法排除了与金属钩相关的并发症，但须注意选择合适的患者。年龄较大或长期吸烟的患者，其肺泡内碳末沉积，肺表面颜色变深，脏层胸膜颜色与亚甲蓝的着色相仿，有时会导致亚甲蓝识别困难。同时也需要保证手术的不间断性，如果注射后不能随即进行手术，则亚甲蓝在肺表面迅速弥散，同样无法识别注射部位^[12]^。

#### 1.1.3 金属钩穿刺联合亚甲蓝染色法

国内詹必成等^[13]^用亚甲蓝与Hookwire联合定位，既克服了亚甲蓝快速弥散、在色素沉着的肺表面不易识别的缺点，又克服了Hookwire容易移位脱落的缺点，从而最大限度地避免了单一运用亚甲蓝或者Hookwire定位失败的可能性。亚甲蓝与Hookwire联合定位后，可以根据定位钢丝很快找到病灶位置，将钢丝提起后，可判断病灶深度，亚甲蓝染色区可清楚显示病灶的范围，沿亚甲蓝染色区与钢丝重叠区域进行楔形切除，有助于快速获得准确的切缘。此方法提高了定位的准确性，但是定位过程相对繁琐，在一定程度上也增加了发生并发症的风险。

#### 1.1.4 溶胶注射法

由于单一亚甲蓝的染色存在一定的缺陷性，1996年Nomori等^[14]^利用不饱和胶原在体内可长时间驻留而没有并发症的特点，将这种不饱和胶原混合亚甲蓝染色，并加入一定量的碘苯六醇制备成溶胶，作为定位标记物。将其在CT引导下经皮穿刺注射到病变周围，这种物质可以在肺组织内驻留较长时间而不弥散，因为胶原含有亚甲蓝，在胸腔镜手术中能够通过肺表面被观察到^[15]^。但较深部位的病变或严重的肺脏碳末沉积的情况下，Nomori对这种技术的可靠性没有作进一步的研究，但因其含有对比剂，估计透视辅助下的胸腔镜手术也能够进行。肺脏的深部病变即使染色后，肉眼有时也难以观察到，同时，微小病变因为太小和部位较深，开胸手术时术者也难以用手触知，Tsuchida等^[16]^为解决这类困难，将琼脂加热溶解后通过穿刺针注射到病变周围，琼脂凝固后变成坚硬的可以触知的结节，达到了定位的目的。但他没有观察琼脂结节在肺内长期驻留的变化，同时也没有尝试此方法是否适合于胸腔镜手术。1998年Choi等^[17]^对硫酸钡、碘油和水溶性对比剂的定位效果进行了对照研究，他们分别应用稀硫酸钡悬液、碘油和水溶性对比剂注射到病变的部位或其周围，然后注射靛蓝胭脂红染色定位针道，随即对患者实施X线透视辅助下的胸腔镜手术，发现碘油无论是从定位效果还是药物准备上，都优于水溶性对比剂和无菌的稀硫酸钡悬液。病理检查发现定位注射物对标本没有明显的污染。虽然碘油在短时间内不会在肺内发生显著的弥散，这种方法同样建议在定位后即刻进行手术。2002年Burdine等^[18]^将放射性锝胶体硫核素在引导下经皮穿刺注射到肺内病灶周围，在实施胸腔镜手术时，使用无菌的γ探头示踪确定放射性核素的区域，然后对此区域行楔形切除。这种方法虽然定位准确，但设备要求特殊，价格昂贵，仅限于非常表浅的病灶。

### 1.2 术中定位法

术中定位方法主要包括超声定位和计算机辅助手术导航系统。

#### 1.2.1 超声定位法

1993年，Shennib等^[19]^在术中肺萎陷后用医学超声技术定位肺微小病变，这是最早对肺部微小病灶进行术中定位的方法报道。因为有时在术前无法确定能否在术中找到病变，因此术前定位的必要性也难以肯定。若术中探查无法触及病灶，则辅以超声定位，可以减少一定数量的不必要的术前定位，是一种无创、简易、经济的方法^[20]^。然而这种方法依然有很大的局限性，一是超声分辨率比较低，难以很好地观察和定位 < 1 cm的病变尤其是纯磨玻璃样病变。二是此操作受到肺组织含气量的影响，要求被检查的肺完全塌陷才能很好地定位，而肺完全塌陷的患者术后扩张不良，哮喘及慢性非阻塞性疾病患者的肺也很难完全塌陷。术中超声对于操作者的依赖性比较高，需要具有丰富经验的操作者。由于这些缺点，此方法正逐渐被淘汰。

#### 1.2.2 导航定位法

2007年陈炜生等^[21]^报道用计算机导航技术对肺部微小病变进行术中定位。计算机辅助手术导航系统把患者术前的影像资料传输到高性能计算机中，重建医学图像模型，在医师的双眼、手术工具及患者的胸部之间建立一个实时的环路，实现手术过程中器械位置的实时显示，这样术者可以通过处理软件在计算机上选择最佳的手术入径，设计最佳手术方案。术中导航系统跟踪手术器械位置并在患者术中影像上实时更新显示，向术者提供手术器械位置的直观、实时信息，引导手术安全进行。手术医生可依靠实时的定位及预设方案的引导在手术中直达病灶。尽管如此，此系统致命缺陷是价格昂贵且操作复杂，因此难以在临床普及。

## 2 定位新方法的思考和实践

随着电子计算机技术、医学影像技术和图像处理技术的飞速发展，在现代CT基础上设计生产的新型CT模拟机，促成了放射治疗技术本质的飞跃。CT模拟机不仅可以像诊断性CT一样为治疗计划提供高质量的横断面影像资料，帮助临床医生精确勾画出肿瘤靶区及危险器官的轮廓，而且能够借助复杂的计算机软件，将计划设计的照射野三维空间分布结果重叠在CT重建的患者解剖资料之中，在相应的激光定位系统的辅助下，实现对治疗条件的虚拟模拟。从肿瘤照射野的定位、治疗计划的设计到治疗计划的模拟实施，CT模拟机的应用贯穿了放射治疗的全过程^[22]^。受到新型放疗CT模拟定位技术的启发，我们设想，能否借助该技术，在术前对患者进行实时病灶定位和皮肤穿刺点模拟标定（包括进针角度及深度），在患者麻醉后依据术前标定位置对病灶进行染色剂注射。如此，一方面可以避免既往术前定位方法中患者和医务人员的额外X线辐照，另一方面能够消除患者清醒穿刺的恐惧，规避术前可能的并发症且丝毫不影响术前常规准备工作。经文献检索术前CT模拟定位结合麻醉后注射染色剂，国内外尚无该方法的研究报道。在上述思路的引导下，我们于2011年下旬进行了初步尝试，取得了较好的结果。基本实施程序如下：①患者选择：肺部周围型微小病灶，CT判断病灶在术中肉眼可能无法窥及或手指难以触知并适合行楔形切除者（图 1）；②CT图像采集：患者在CT扫描机下行胸部CT薄层扫描（层厚3 mm），体位的选择要求患者感到舒适、易于保持、方便重复^[23]^。扫描前根据已掌握的临床资料对可能的穿刺方向做出判断，调整好患者的位置。嘱患者尽量保持平静呼吸，减少呼吸动度的影响。利用激光定位仪在皮肤做金属标记点，确定原始坐标系，行CT扫描，将采集的图像传输到计划系统；③定位计划并皮肤标记：使用治疗计划计算机系统进行图象三维重组、勾画靶区，根据三维结构及靶区位置及临床治疗要求，设计者可给出射线的方向，根据软件系统可得到射线在皮肤的位置到病灶中心的距离。射线在皮肤的投射点即为皮肤进针穿刺点，射线方向即为进针角度，皮肤的位置到病灶中心的距离即为进针深度。在治疗计划系统提供的软件帮助下，射线在体表皮肤的任意变化均可提供相应的角度及深度值。这样可以实时选择合适的皮肤进针点，使其到病灶中心的路径最短，且避开骨性结构、血管及神经。进针角度宜尽量垂直或平行于水平面，可减少穿刺操作时因进针角度不易控制而出现误差。计划制定好后，让患者重复CT扫描时的体位，通过计算机控制机架和治疗床的运动，使激光十字照射在先前治疗计划系统确定的皮肤进针点，并用标记笔标记（图 2）；④手术室定位操作：患者在全身麻醉后，人工控制患者体位尽量保持与做CT扫描时一致。根据之前做的皮肤标记点及治疗计划系统提供的进针角度和深度数据，穿刺病灶并注射亚甲蓝标记（图 3）；⑤即刻行胸腔镜或开放手术，术中根据肺表面亚甲蓝着色位置触摸查找病灶（图 4），行肺楔形切除术，根据术中冰冻病理决定进一步处理方案（图 5）。

**1:**
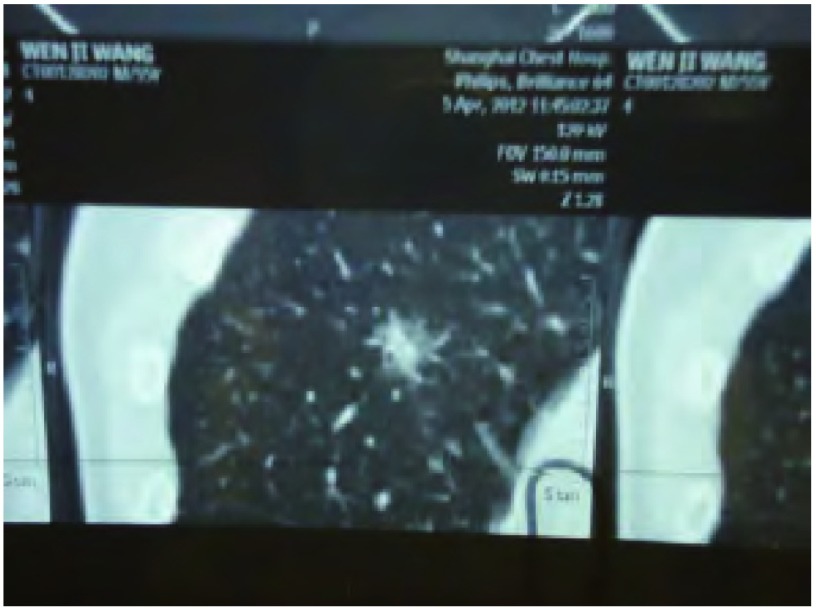
胸部CT病灶 The lesion of CT scan. CT: computed comography.

**2:**
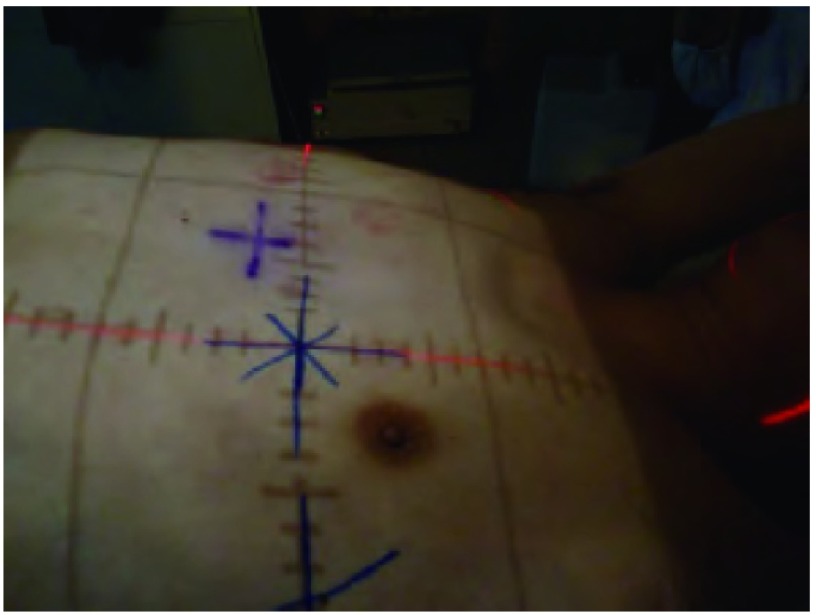
术前模拟定位 Preoperative simulation positioning

**3:**
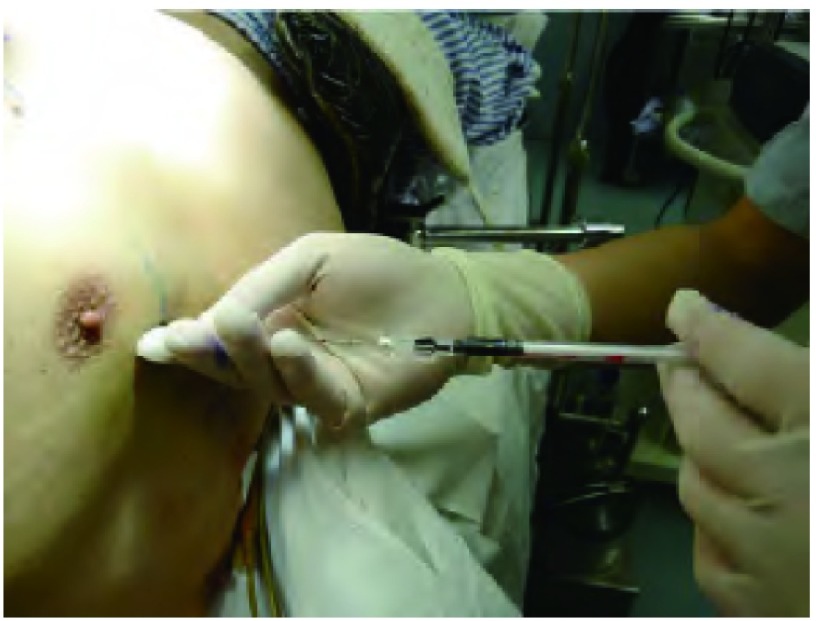
注射亚甲蓝 Inject methylene blue

**4:**
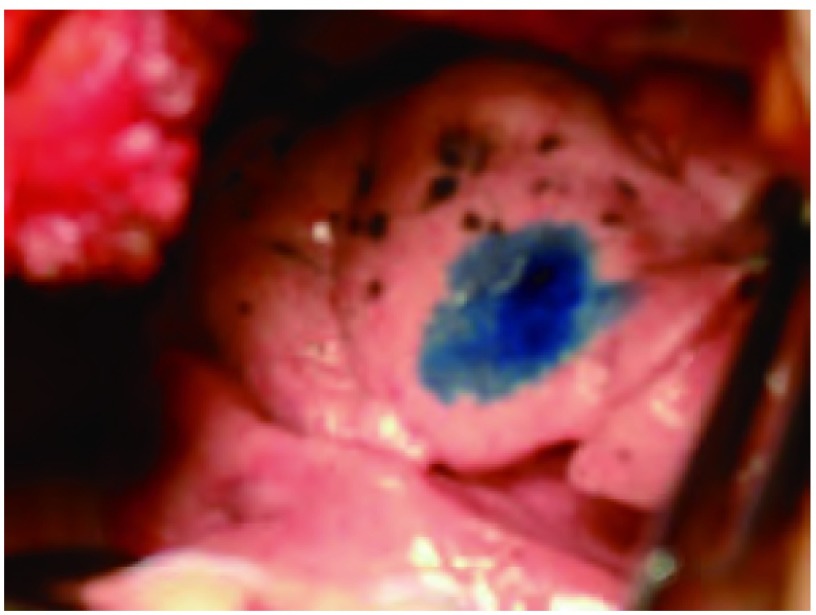
术中所见染色 Intraoperative dyeing

**5:**
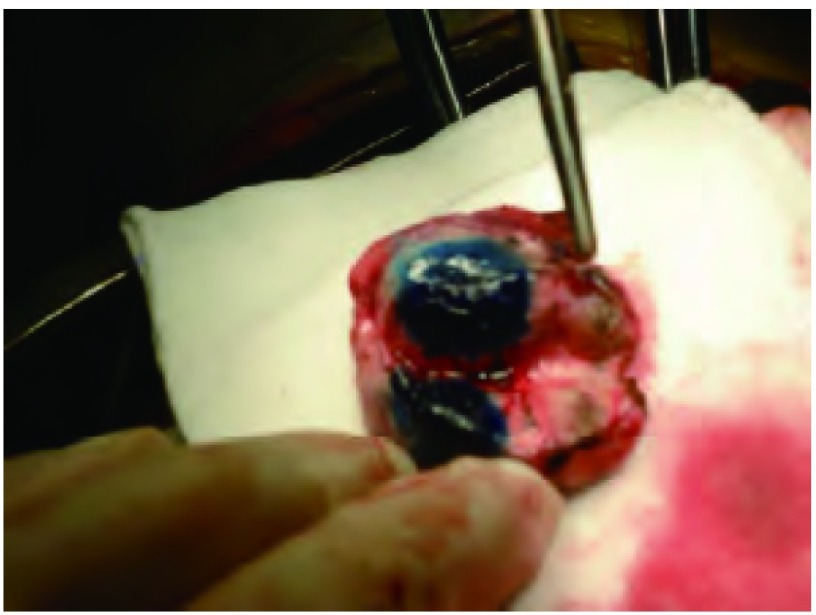
切开病灶 Cut the lesion

## 3 结语

综上所述，对于周围型肺部微小病灶，现有术前定位方法各有利弊，有待进一步探索更为安全、简易、经济、准确的方法。应用CT模拟机术前对病灶虚拟定位继以麻醉后注射亚甲兰的方法，能够避免医务人员承受辐射，消除患者接受胸部穿刺的恐惧和穿刺相关的并发症，定位后便能即刻行剖胸手术，凡此种种，均显示了令人兴奋的明显优势，作者的初步尝试获得了比较满意的结果，值得进一步验证其定位的准确性和实用性。
